# Comparison of two types of the triple incision technique in the treatment of patients with locally advanced vulvar cancer

**DOI:** 10.7150/ijms.49804

**Published:** 2020-09-16

**Authors:** Ying Ma, Wei-feng Liang, Chang-hao Liu, Zhong-qiu Lin, Miao-fang Wu, Jing Li

**Affiliations:** 1 Department of Obstetrics and Gynecology, the First Affiliated Hospital of Dalian Medical university, Dalian, 116011, People's Republic of China.; 2 Department of Gynecology and Obstetrics, Qilu Hospital (Qingdao), Cheeloo College of Medicine, Shandong University, Qingdao, 266035, People's Republic of China.; 3 Department of Gynecologic Oncology, Sun Yat-sen Memorial Hospital, Sun Yat-sen University, Guangzhou, 510120, People's Republic of China.

**Keywords:** vulvar cancer, triple-incision technique, complication, survival

## Abstract

**Objective:** In 2012, we proposed and described a modified triple incision technique (MTIT) for vulvar cancer patients with locally advanced disease. The MTIT has undergone a series of modifications, and a modified MTIT (M-MTIT) has been developed. The purpose of this study was to introduce the M-MTIT and compare it with the MTIT.

**Study design:** This was a retrospective cohort study. Fifty-seven vulvar cancer patients with clinical stage T2 (≥ 4 cm) or T3 disease were included. Of these patients, 28 underwent the MTIT and 29 underwent the M-MTIT. Data on surgery-related complications and survival outcomes were compared.

**Results:** Patients who were treated with the M-MTIT developed significantly less surgery-related morbidities than patients treated with the MTIT (24.1% vs. 60.7%, *P* = 0.005). Wound breakdown was the most common complication in our cohort, which occurred less frequently in the M-MTIT group than in the MTIT group (10.3% vs. 35.7%, *P* = 0.022). Multivariate logistic regression analysis identified the M-MTIT as an independent predictor of a reduced risk of wound breakdown. The incidence of other complications, including lymphedema, wound infection and cellulitis, was lower in the M-MTIT group than in the MTIT group; however, the differences did not reach statistical significance. The median follow-up time of this study was 33 months. Kaplan-Meier survival graphs did not show significant differences in recurrence-free survival or overall survival between the two groups.

**Conclusions:** The M-MTIT correlates with lower morbidity rates than the MTIT and does not compromise oncological safety. The M-MTIT can be considered a safe and feasible option for vulvar cancer patients with locally advanced disease.

## Introduction

For patients with vulvar cancer, radical excision of the tumor with inguinofemoral lymph node dissection (ILND) is a major extirpative procedure 1-3. Although the efficacy of this treatment is good, morbidity following surgery can be substantial. To reduce complication rates, considerable efforts have been made in recent years. Introduction of the sentinel-node procedure was one of the most promising advances and has been validated as a safe alternative to ILND for patients with early-stage disease (T1 or T2 < 4 cm) 4, 5. However, for patients with more advanced disease, en bloc radical vulvectomy with bilateral ILND remains the standard surgical procedure 1, 2. In 2012, we reported a modified triple incision technique (MTIT) and proposed it for vulvar cancer patients with locally advanced disease 6. While data from the study suggested that the MTIT could be safe, feasible and tolerable, there is still room for improvement. Since 2009, the original MTIT has undergone several modifications. The aims of the present study were (1) to evaluate whether the modified MTIT (M-MTIT) is superior to the MTIT in terms of postoperative morbidity and (2) to assess whether the M-MTIT is equal to the MTIT regarding oncological outcomes.

## Materials and methods

### Patients

Institutional Review Board approval was obtained from the Sun Yat-sen Memorial Hospital (IRB Number: SYSEC-KY-KS-2020-045). Data were collected retrospectively from records of patients who underwent the M-MTIT or MTIT between January 2004 and December 2016. Tumors were classified according to the American Joint Committee on Cancer Staging System (1992). Our operative criteria were as follows: histologically confirmed invasive squamous cell carcinoma (stromal invasion > 1 mm); clinical stage T2 [≥ 4 cm] or T3; American Society of Anesthesiologists (ASA) score ≤ 3. Patients who had clinically palpable lymph nodes or evidence of distant metastasis and patients with a history of previous vulvar and/or pelvic radiation therapy were excluded.

**Figure 1:**
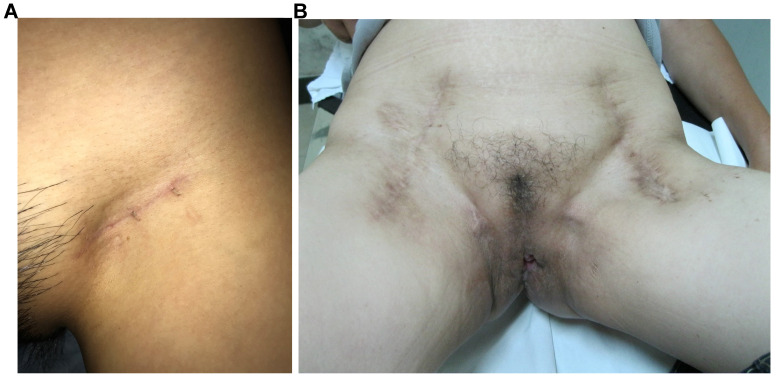
Appearance on the groin. (**A**) M-MTIT groin incision 8 months post-operative. (**B**) MTIT groin incision 12 months post-operative.

All vulvar cancer patients with clinical stage T2 [≥ 4 cm] or T3 disease, comprehensive preoperative evaluation was conducted by a multidisciplinary team, which consisted of two gynecologic oncologists, one radiologist, one urologist and one plastic surgeon 7, 8. For patients who were considered as poor candidates for surgery, concurrent chemoradiotherapy was recommended. All surgical procedures were performed by the same gynecologic oncology team. All patients received inguinal and femoral node dissection, which included superficial inguinal nodes and deep groin nodes. An equal extent of lymphadenectomy was employed in the MTIT and M-MTIT, and the great saphenous vein was preserved during the ILND procedure. The MTIT was performed as previously described 6. Following the inguinofemoral lymphadenectomy, all patients received a modified radical vulvectomy. During the process of vulvectomy, the inner incision was deepened down to the inferior fascia of the urogenital diaphragm, while the outer incision was undermined laterally to separate the subcutaneous tissue of the skin bridge from the fat pad of the lateral thigh. Finally, the groin node-bearing fatty pad was removed together with the complete radical vulvectomy specimen. In case of a large defect of the vulva, plastic surgery reconstruction involving skin-flaps was performed immediately by experienced plastic surgeons 9. The M-MTIT differed in three respects from the original MTIT. First, an inguinal incision of 3-5 cm that was parallel to the inguinal ligament was used in the M-MTIT (**Figure 1A**); while a midline vertical inguinal incision of 8-10 cm was employed in the MTIT (**Figure 1B**). Second, radical excision of the vulvar tumor was performed with at least 1 cm clinically clear surgical margins in the M-MTIT, while the minimal macroscopic resection margin was 2 cm in the MTIT. Third, all patients treated with the M-MTIT received negative pressure wound therapy on groin incisions, while patients undergoing the MTIT received standard-of-care dressing. The duration of negative pressure therapy was five to seven days.

We used the same criteria as Rouzier for reporting postsurgical morbidity 10. The adjuvant treatment following surgery was performed according to the National Comprehensive Cancer Network (NCCN) guidelines 1, and indications for adjuvant chemoradiotherapy on the groin and pelvis were as follows: close or positive surgical margins, lymphovascular space invasion (LVSI), lymph node metastasis and/or extracapsular lymph node involvement. Following initial treatment, patients underwent follow-up examinations every 3 months for the first 2 years, every 6 months for the next 3 years, and every year thereafter. Follow-up visits included an interview, palpation of the groins and a gynecological examination. Imaging studies, including computed tomography (CT), magnetic resonance imaging (MRI) and positron emission tomography (PET)/CT, were performed at the discretion of the treating physician. When tumor recurrence was suspected based on clinical findings or imaging studies, biopsies of the suspicious lesions were performed on a case-by-case basis.

### Statistical analysis

We used STATA 12.0 (StataCorp, College Station, TX, USA) to perform all the statistical analyses. Baseline patient characteristics were compared between groups using Student's *t* test or the Mann-Whitney *U* test for continuous variables and the* χ*^2^ test or Fisher's exact test for categorical variables as appropriate. Multivariate logistic regression was used to determine independent risk factors for wound breakdown. All variables with significance at* P* < 0.30 in the univariate analysis were considered candidates in the final model. This relatively liberal cutoff was chosen due to the small sample size that may have a risk of type II error with a lower cutoff. Survival curves were computed with the Kaplan-Meier method, and the significance of each survival difference was determined with the log-rank test. All statistical tests were two-sided, and a two-tailed *P*-value <0.05 was considered statistically significant.

## Results

### Clinical and pathological variables

**Table 1** demonstrates the characteristics of the 57 patients who were included in the analysis. In our patient cohort, 28 (49.1%) underwent the MTIT and 29 (50.9%) underwent the M-MTIT. The majority (73.7%) had T2 > 4 cm disease, the median age at diagnosis was 58 years (range: 30-80 years), and the median body mass index (BMI) was 22.1 kg/m^2^ (range: 15.2-28.4 kg/m^2^). Fourteen (24.6%) patients had comorbidities that included hypertension, diabetes mellitus, heart disease, chronic obstructive pulmonary disease and systemic lupus erythematosus. No significant difference was noted between the two groups with respect to demographic characteristics and type of surgery.

### Treatment-related variables

As deemed necessary by the primary surgeon to achieve adequate surgical margins, a colostomy, partial vaginectomy and partial urethral resection were performed in three (5.2%), four (7.0%) and nine (15.8%) patients, respectively. Among the nine patients receiving urethral resection, three underwent a urethral resection of 1.0 cm, two underwent a urethral resection of 1.5 cm, and one underwent a urethral resection of 2 cm. The length of the removed urethra was not recorded in the other three patients.

Operative data and histopathological findings are listed in **Table 2.** The median number of nodes removed was 12 (range: 8-17) and did not differ between the two groups. Following surgery, no patient experienced skin necrosis or urine incontinence. The median postoperative hospital stay was 30 days (range: 7-54 days) and was shorter in the M-MTIT group than in the MTIT group (25 days [range: 7-49 days] vs. 36 days [range: 24-54 days], respectively, *P* < 0.0001). Surgery related complications were noted in 24 (42.1%) patients and 11 of them developed more than two complications. Surgical outcomes are summarized in **Table 2.** Compared with patients in the MTIT group, fewer patients in the M-MTIT group had morbidities (24.1% vs. 60.7%, *P* = 0.005). Wound breakdown was observed in 13 patients (22.8%) and was less often observed in patients receiving the M-MTIT than in those receiving the MTIT (10.3% vs. 35.7%, *P* = 0.022). The incidence of other major complications, including lymphedema (34.5% vs. 42.9%, *P* = 0.516), wound infection (3.5% vs. 17.9%, *P* = 0.076) and cellulitis (3.5% vs. 10.7%, *P* = 0.283), was lower among patients undergoing the M-MTIT; however, the differences did not reach statistical significance.

Because wound breakdown was the most common short-term complication of the entire cohort, we conducted regression analysis to detect independent predictors associated with it. The results are listed in **Table 3.** On the univariate analysis, only the inguinal incision type was significantly (odds ratio [OR] = 0.21, 95% confidence interval [CI] [0.05, 0.86], *P* = 0.030) associated with the risk of wound breakdown. On the multivariate analysis, which incorporated all variables with *P* < 0.30 on the univariate analysis, the type of inguinal incision remained independently associated with the risk of wound breakdown (OR = 0.18, 95% CI [0.04, 0.83], *P* = 0.028).

### Survival analysis

**Figure 2:**
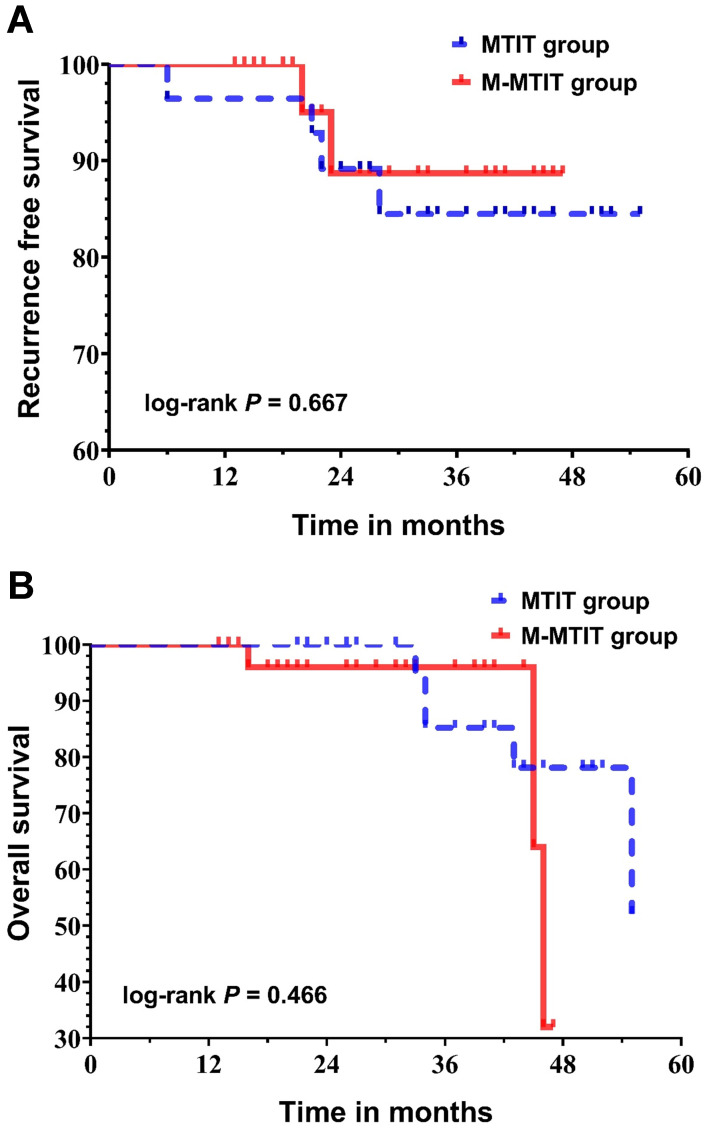
Kaplan-Meier curves of recurrence-free survival (**A**) and overall survival (**B**).

The median follow-up time was 33 months (range: 13-55 months) for the entire cohort, and no patient was lost to follow-up. As shown in **Table 4**, four patients in the MTIT group (6, 21, 22 and 28 months after primary surgery) and two patients in the M-MTIT group (20 and 23 months after primary surgery) experienced tumor recurrence. Of the six patients who developed recurrence, skin abridge failure was noted in three, and all of the lesions were successfully salvaged with a local re-resection. Distant recurrence was documented in three patients who underwent tumor-targeted radiotherapy and systemic chemotherapy but ultimately died of disease. Eight deaths were recorded, five (62.5%) of which were a result of medical comorbidities, including pulmonary infection, respiratory failure, heart failure, stroke and renal failure. **Figure 2** demonstrates the survival curves for recurrence-free survival (RFS) and overall survival (OS). The estimated 1-, 2-, and 3-year RFS rates for patients receiving the MTIT were 92.9%, 89.1%, and 84.5%, respectively, compared with 100%, 88.7%, and 88.7%, respectively, for those receiving the M-MTIT. The estimated 1-, 2-, and 3-year OS rates for patients receiving the MTIT were 100%, 100%, and 85.2%, respectively, compared with 100%, 96.0%, and 96.0%, respectively, for those receiving the M-MTIT. RFS (unadjusted hazard ratio [HR] = 0.69, 95% CI [0.13, 3.78]; log-rank *P* = 0.667) was similar in the two groups, as was OS (unadjusted HR = 1.76, 95% CI [0.38, 8.11]; log-rank *P* = 0.466).

## Discussion

For vulvar cancer patients with locally advanced disease (clinical stage T2 [≥ 4 cm] or T3), en bloc radical vulvectomy with bilateral ILND remains the optimal therapeutic option 1-3. The rate of complications following this treatment is significant, occurring in up to 85% of patients 11. To reduce surgery-related morbidity, several modified surgical methods have been proposed. Although the MTIT is less morbid than the traditional en bloc approach, a considerable portion of patients still suffer from postoperative complications 6. Thus, we proposed the M-MTIT, which can be considered a modification of the MTIT, and we made comparisons between the M-MTIT and MTIT. Our data suggest that the M-MTIT is superior to the MTIT.

In the present study, wound breakdown was the most common postsurgical complication with an incidence of 22.8%, which is consistent with previous reports. However, patients in the M-MTIT group had a significantly lower incidence of wound breakdown than patients in the MTIT group (10.3% vs. 35.7%, *P*=0.022). This finding was further strengthened by the regression analysis, where the M-MTIT was identified to be independently associated with a decreased risk of wound breakdown (OR = 0.18, 95% CI [0.04, 0.83], *P* = 0.028). Among previous studies regarding the triple incision technique, Rouzier's retrospective study examined the largest sample size to date: 194 vulvar cancer patients 10. The authors found that the incidence of wound breakdown is influenced by the extent of lymphadenectomy 12, 13, and the highest incidence (38.3%) was documented in patients receiving ILND via the traditional triple incision technique. The extent of ILND and criteria to define complications in the current study were the same as those described by Rouzier et al. 10. However, we found that only 10.3% of the patients in the M-MTIT group experienced wound breakdown. By comparing the M-MTIT with the MTIT and Rouzier's data 10, we believe that the M-MTIT can reduce the risk of wound breakdown. This benefit may be attributed to the small inguinal incision used in the M-MTIT and the use of negative pressure wound therapy 14-16.

Other surgery-related complications, including lymphedema (34.5% vs. 42.9%, *P*=0.516), wound infection (3.5% vs. 17.9%, *P*=0.076) and cellulitis (3.5% vs. 10.7, *P*=0.283), were less likely to occur in patients treated with the M-MTIT than in patients treated with the MTIT; however, the differences did not reach statistical significance. Despite this finding, compared with the reported incidence of lymphedema (47%) and cellulitis (29.1%) by Rouzier 10, the M-MTIT is less morbid. The development of and cellulitis is related to the extent of lymphadenectomy 11, 17, and 10 nodes from a bilateral dissection can be considered radical for vulvar cancer patients 18, 19. In the present study, the median numbers of resected nodes in the M-MTIT and MTIT groups were 13 and 12, respectively (*P*=0.270). Therefore, the lower morbidity associated with the M-MTIT does not compromise the radical tumor clearance of lymphadenectomy. The great saphenous is an important route of lower extremity lymphovascular circulation. Preservation of the great saphenous during lymphadenectomy has been reported as an effective method to reduce the risk of lymphedema and infection 10, 20, which could explain the differences between our data and others.

Locally advanced vulvar cancer is associated with a significant risk of local recurrence, and the tumor margin status has been validated as a significant prognostic factor 1, 2, 21. To decrease the risk of local recurrence, most guidelines recommend a histological tumor-free margin of at least 8 mm 1, 3, 22-24, which corresponds to a surgical margin of 1-2 cm 25. There is evidence showing 0% recurrence rates for > 8 mm margins and 47% when the margins are ≤ 8 mm 3, 25, 26. However, a recent multicenter cohort study by the Francogyn Study Group, where 112 vulvar cancer patients who received treatment in four French university hospitals were included, did not find that tumor-free margin distance had survival influence 26. In Micheletti's study 27 which included 114 patients, the minimum histological margin distance that conferred long-term oncological safety was 5 mm. Of note, this study reviewed data from 1981 to 2014, during which time the management of vulvar cancer has changed considerably. The theory of ontogenetic cancer fields could be a plausible explanation for the inconsistent findings in previous studies 28, 29. Collectively, in light of current evidence, there is no convincing data supporting that pathologic margins < 8 mm is safe. The width of the surgical margin (1 cm vs. 2 cm) was relatively narrow in the M-MTIT group; however, this did not result in an increased incidence of microscopic positive margins (3.5% vs. 3.6%, *P*=0.980). Based on our findings and available evidence, we believe that a macroscopic tumor-free margin should be at least 1 cm 1-3, 22-24.

The median follow-up time of our study was 33 months, which allowed us to observe most cases of recurrence 30. We did not find any survival difference between the two groups. Additionally, survival data in the current study were comparable to those in other studies 10, 31. These findings suggest that oncological safety can be ensured with the M-MTIT.

Of note, patients enrolled in this study, with the median age of 58 years, were younger than in white patient populations in previous studies 32, 33, but the median age was consistent with that reported in similar studies involving Chinese patients 34, 35. Additionally, a severe reduction of OS was noted after 48 months of surgery. Some of our patients were older and some had serious complications at diagnosis, which may explain why mortality increased significantly over time. Furthermore, we acknowledge that the present study had some limitations. First, this study is limited by biases related to its retrospective design. In addition, vulvar cancer is a rare disease. Although we pooled 12 years of data, the sample size of this work is only moderate for survival analyses, and it is difficult to identify more complication-related risk factors. Additionally, many Chinese patients favor a longer hospital stay because they believe it assures a better recovery 6. Thus, the length of hospital stay may not serve as an objective factor. Third, since patients in the M-MTIT group received negative pressure therapy following surgery, there were differences in wound care between the groups. This could have resulted in biased results when we assessed the wound complications. Finally, although the two technologies differ in three main ways that include the width of the surgical margin, surgical incisions and the utility of negative pressure wound treatment, the specific influence of each of these variables on the final outcomes could not be clarified.

In summary, our study suggests that the M-MTIT correlates with lower morbidity rates than the MTIT and other traditional triple incision techniques and does not compromise oncological safety. We believe that the M-MTIT should be considered a safe and feasible option for vulvar cancer patients with locally advanced disease. Further investigations are merited to validate our conclusions.

## Figures and Tables

**Table 1:** Patient characteristics

Variables	MTIT (n=28)	M-MTIT (n=29)	*P* value
Age (years), median (range)	57.5 (30-75)	58.0 (32-80)	0.367
BMI (kg/m²), median (range)	22.4 (15.2-28.4)	21.4 (16.7-26.2)	0.515
**Smoking, n (%)**			
Never	24 (85.7)	26 (89.7)	0.158
Former	4 (14.3)	1 (6.9)	
Current	0 (0)	2 (3.5)	
**HPV infection, n (%)**			
No	0 (0)	1 (3.5)	0.861
High risk	11 (39.3)	14 (48.3)	
Low risk	6 (21.4)	6 (26.7)	
Unreported	5 (17.9)	3 (10.3)	
Comorbidities	6 (21.4)	5 (17.2)	
Diameter of primary tumor (cm), median (range)	5.5 (4-8)	5.5 (4-8)	0.493
**Grade of primary tumor, n (%)**			
G1-2	20 (71.4)	20 (69.0)	0.839
G3	8 (28.6)	9 (31.0)	
**Clinical stage of the primary tumor, n (%)**			
T2>4cm	20 (71.4)	22 (75.9)	0.704
T3	8 (28.6)	7 (24.1)	
**Patients with comorbidities, n (%)**	6 (21.4)	8 (27.6)	0.589
Hypertension	2 (7.1)	3 (10.3)	
Diabetes mellitus	1 (3.6)	2 (6.9)	
Heart disease	2 (7.1)	2 (6.9)	
COPD	1 (3.6)	0 (0)	
SLE	0 (0)	1 (3.5)	

BMI, body mass index; COPD, Chronic obstructive pulmonary disease; HPV, human papillomavirus; MTIT, modified triple incision technique; M-MTIT, modified MTIT; SLE, systemic lupus erythematosus.

**Table 2:** Surgical characteristics and incidence of post-operative complications

Variables	MTIT (n=28)	M-MTIT (n=29)	*P* value
Operative time (min), median (range)	141 (70-265)	150 (70-295)	0.648
Estimated blood loss (ml), median (range)	50 (10-300)	60 (10-200)	0.550
Colostomy, n (%)	2 (7.1)	1 (3.5)	0.975
Vulvovaginal reconstruction, n (%)	6 (21.4)	6 (20.7)	0.945
Partial vaginectomy, n (%)	2 (7.1)	2 (6.9)	0.630
Partial urethral resection, n (%)	4 (14.3)	5 (17.2)	0.954
No. of lymph nodes removed, median (range)	12 (9-16)	13 (8-17)	0.270
Blood transfusion, n (%)	4 (14.3)	1 (3.5)	0.148
Positive margins, n (%)	1 (3.6)	1 (3.5)	0.980
Positive nodes, n (%)	8 (28.6)	9 (31.0)	0.839
LVSI, n (%)	6 (21.4)	4 (13.8)	0.449
Postoperative concurrent chemoradiotherapy, n (%)	9 (32.1)	9 (31.0)	0.928
Lymphedema, n (%)	12 (42.9)	10 (34.5)	0.516
**Wound breakdown, n (%)**	10 (35.7)	3 (10.3)	0.022
vulvar wound	0	0	
groin wound	10 (35.7)	3 (10.3)	0.022
**Wound infection, n (%)**	5 (17.9)	1 (3.5)	0.076
vulvar wound	3 (10.7)	1 (3.5)	0.579
groin wound	2 (7.1)	0	0.143
Cellulitis, n (%)	3 (10.7)	1 (3.5)	0.283
DVT, n (%)	1 (3.6)	1 (3.5)	0.980
Urine incontinence, n (%)	0 (0)	0 (0)	
Postoperative hospital stay (days), median (range)	36 (24-54)	25 (7-49)	<0.0001

DVT, deep vein thrombosis; LVSI, lymphovascular space invasion; MTIT, modified triple incision technique; M-MTIT, modified MTIT.

**Table 3:** Univariate and multivariate analysis of factors associated with wound breakdown

	Univariate analysis			Multivariate analysis		
	OR	95% CI	*P* value	OR	95% CI	*P* value
Types of triple incision(M-MTIT vs. MTIT)	0.21	0.05-0.86	0.030	0.18	0.04-0.83	0.028
Age (years)	0.99	0.94-1.04	0.576			
BMI (kg/m^2^)	1.04	0.83-1.29	0.741			
Operative time (min)	1.00	0.99-1.02	0.422			
Estimated blood loss (ml)	1.01	1.00-1.02	0.063	1.01	1.00-1.02	0.072
Comorbidity (yes vs. no)	1.51	0.38-5.96	0.556			
Stage (T3 vs. T2>4cm)	2.13	0.57-7.96	0.263	1.45	0.30-7.01	0.641
Diameter (cm)	1.38	0.75-2.51	0.297	1.23	0.61-2.49	0.568

BMI, body mass index; CI, confidence interval; MTIT, modified triple incision technique; M-MTIT, modified MTIT; OR, odds ratio.

**Table 4:** Pattern of recurrence and deaths by treatment arm

	MTIT (n=28)	M-MTIT (n=29)
Time of follow-up, median (range) (months)	41 (21-55)	27 (13-47)
No. of patients with recurrence	4	2
**Site of recurrence**		
Skin abridge	2	1
Lung	1	1
Bone	1	0
No. of death	5	3
**Cause of death**		
Tumor recurrence	2	1
Non-cancer-related causes	3	2

MTIT, modified triple incision technique; M-MTIT, modified MTIT.
